# Editorial: New Perspectives on Domestic Violence: From Research to Intervention

**DOI:** 10.3389/fpsyg.2019.00641

**Published:** 2019-03-28

**Authors:** Luca Rollè, Shulamit Ramon, Piera Brustia

**Affiliations:** ^1^Department of Psychology, University of Turin, Turin, Italy; ^2^School of Health and Social Work, University of Hertfordshire, Hertfordshire, United Kingdom

**Keywords:** domestic violence, intimate partner abuse, intimate partner violence (IPV), gender violence against women, same sex intimate partner violence, systematic review, perpetrator and victim of violence, perpetrator

In a document dated June 16th 2017, the United States Department of Justice stated that Domestic Violence (DV) has a significant impact not only on those abused, but also on family members, friends, and on the people within the social networks of both the abuser and the victim. In this sense, children who witness DV while growing up can be severely emotionally damaged. The European Commission (DG Justice) remarked in the Daphne III Program that 1 in 4 women in EU member states have been impacted by DV, and that the impact of DV on victims includes many critical consequences: lack of self-esteem, feeling shame and guilt, difficulties in expressing negative feelings, hopelessness and helplessness, which, in turn, lead to difficulties in using good coping strategies, self-management, and mutual support networks. In 2015 the EU Agency for Fundamental Rights affirmed that violence against women can be considered as a violation of human rights and dignity. Violence against women exists in each society and it can be related to any social, economic and cultural status and impact at the economic level. It includes physical, sexual, economic, religious, and psychological abuse.

Although men experience domestic violence by women, the rate of DV among women is much higher than that of men, especially in the category of being killed due to DV.

Recent studies have shown that between 13 and 61% of women (15–49 years old) report to have been physically abused at least once by an intimate partner. Domestic Violence takes place across different age groups, genders, sexual orientations, economic, or cultural statuses. However, DV remains largely under-reported due to fear of reprisal by the perpetrator, hope that DV will stop, shame, loss of social prestige due to negative media coverage, and the sense of being trapped with nowhere to go:

Hence, it is estimated that 90% of cases of DV continue to be identified as a non-denounced violence.

The aim of this Special Issue of Frontiers of Psychology is to gather updated scientific and multidisciplinary contributions about issues linked to domestic violence, including intimate partner violence (IPV). We encouraged contributions from a variety of areas including original qualitative and quantitative articles, reviews, meta-analyses, theories, and clinical case studies on biological, psycho-social and cultural correlates, risk and protective factors, and the associated factors related to the etiology, assessment, and treatment of both victims and perpetrators of DV.

We hope that this Special Issue will stimulate a better informed debate on Domestic Violence, in relation to its psychosocial impact (in and outside home, in school, and workplace), to DV prevention and intervention strategies (within the family and in society at large), in addition to specific types of DV, and to controversial issues in this field as well.

The Special Issue comprises both theoretical reviews and original research papers. 7 research papers, 6 reviews (policy and practice review, systematic review, review and mini-review) and 1 methodological paper are included.

The first section comprises 2 systematic review and 3 original research papers focused on factors associated with Domestic Violence/Intimate Partner Violence/feminicide. Velotti et al. conducted a systematic review focused on the role of the attachment style on IPV victimization and perpetration. Several studies included failed to identify significant associations. The authors suggest to consider other variables (e.g., socioeconomic condition) that in interaction with attachment styles could explain the differences found between the studies. Considering the clinical contribution that these findings can provide to the treatment of IPV victims and perpetrators, future studies are needed. From a systematic review conducted by Gerino et al. focused on IPV in the “golden age” (old age), economic and educational conditions, younger age (55–69), membership in ethnic minorities, cognitive and physical impairment, substance abuse, cultural and social values, sexism and racism, were found as risk factors; depression emerged as risk factor and consequence of IPV. However, social support was identified as main protective factor. Also help-seeking behaviors and local/national services had a positively impact the phenomenon. Furthermore, the role of the parental communication was highlighted (Rios-González et al.) In that mothers encourage daughters to engage in relationship with ethical men, while removing from their representation attractive features and enhancing the double standard of viewing ethical man as unattractive vs. violent and attractive man. Fathers' communication directed toward young boys supports the dominant traditional masculinity, objectifying girls and emphasizing chauvinist values. These communicative dynamics impact males' behavior and females' choice of the partner while increasing the attraction toward violent men, and thus influencing the risk to be involved in IPV episodes.

Furthermore, factors associated with multiple IPV victimization by different partners were identified. From the study of Herrero et al., experiencing child abuse emerged as a main predictor (“conditional partner selection process”). Similarly, adult victimization perpetrated by other than the intimate partner influences multiple IPV episodes. Moreover, this phenomenon is more frequent among younger women and those with lower income satisfaction. Length of relationship and greater psychological consequences to previous IPV are positively associated with multiple IPV episodes, while previous physical abuse is negatively related with subsequent victimization. The risk of multiple IPV episodes is reduced in countries with greater human development, suggesting the role of structural factors.

Regarding reasons of feminicide, passion motives assume the main role, followed by family problems, antisocial reasons, predatory crimes that comprise sexual component, impulsivity and mental disorders. The risk of overkilling episodes is higher when the perpetrator is known by the victim and when the murder is committed for passion reasons (Zara and Gino).

The second section includes papers focused on IPV/DV in particular contexts (one research paper, two reviews). Within separated couples, where conflicts are common, both men and women experience psychological aggression. However, some particularities emerged: women started to suffer of several kinds of psychological violence that was aimed to control (complicating the separation process), dehumanize and criticize them. Men report only few forms of violence experienced (likely due to the men's social position that narrows their disclosure opportunity), which mainly concern the limitation of the possibility to meet children (Cardinali et al.). Regarding same-sex couples (Rollè et al.), both similarities and differences in comparison with heterosexual couples emerged. IPV among LGB people is comparable or even higher than heterosexual episodes. Unique features present in same-sex IPV concern identification and treatment aspects, mainly due to the absence of solutions useful in addressing obstacles to help-seeking behaviors (related to fear of discrimination within LGB community), and the limitation of treatment programs tailored to the particularities of the LGB experience. Similarly, within First Nation's communities in Canada, IPV is a widespread phenomenon. However, the lack of preventing programs and the presence of intervention solutions that fail to address its cultural origins, limit the reduction of the problem and the recovery of victims. Klingspohn suggests the development of interventions capable to guarantee cultural safety and consequently to reduce discrimination and marginalization that Aboriginal people experience with mainstream health care system and which limit help-seeking behaviors.

The third section comprises two reviews and one research paper concerned with the impact of Intimate Partner and Domestic Violence. The systematic review conducted by Onwumere et al. highlighted the financial and emotional burden that violence perpetrated by psychotic patients entails for their informal carers (mainly close family relatives). Moreover, the authors identified within the studies included positive association between victimization and trauma symptoms, fear, and feeling of powerless and frustration.

Among people who suffered of Domestic Violence with a romantic or non-romantic partner who became their stalker, stalking victimization entails physical and emotive consequences for both male and female victims. Females suffered more than males of depressive and anxiety symptoms (although for both genders symptoms were minimal), while males experienced more anger. Furthermore, both genders adopted at least one “moving away” strategy in coping with stalking episodes, and the increasing of stalking behaviors determined a reduction in coping strategies use. This latter finding is likely to be due to the distress experienced (Acquadro Maran and Varetto).

Children abuse—which occurs often in Domestic Violence—results in emotional trauma as well as physical and psychological consequences that can negatively impact the learning opportunities. The school staff's ability to identify abuse signals and to refer to professionals constitute their main role. However, lack of skills and confidence among teachers regarding this function emerged, and further training for the school staff to increase support provided to abused children is needed (Lloyd).

Lastly, the fourth section includes two papers (one review and one methodological paper) that provide information on intervention and prevention programs and one research paper which contributes to the development and validation of the Willingness to Intervene in Cases of Intimate Partner Violence Against Women (WI-IPVAW) Scale. Gracia et al. The instrument demonstrated—both in the long and in its short form—high reliability and construct validity. The development of WI-IPVAW can contribute to the evaluation of the t role that can be played by people who are aware of the violence and understand attitudes toward IPV that can influence perpetrator's behavior and victim disclosure. The origin of violence within intimate relationship during adolescence calls for the development of preventive programs able to limit the phenomenon. The mini-review conducted by Santoro et al. highlighted the necessity to consider the relational structure where women are involved (history of poly-victimization re-victimization), and the domination suffered according to the gender model structured by the patriarchal context. Moreover, considering that violence can occur after separation or divorce, requires in child custody cases the evaluation of parenting and co-parenting relationship. This process can provide an opportunity to assess and treat some kind of violent behavior (Conflict-Instigated Violence, Violent Resistance, Separation-Instigated Violence). According to these consideration, Gennari et al. elaborated a model for clinical intervention (relational-intergenerational model) useful to address these issues during child custody evaluation. The model is composed of three levels aimed at understanding intergenerational exchange and identify factors that contribute to safeguard family relationship. This assessment process allows parents to reflect on information emerged during the evaluation process and activate resources useful to promote a constructive change of conflict dynamics and violent behaviors.

## Author Contributions

All authors listed have made a substantial, direct and intellectual contribution to the work, and approved it for publication.

### Conflict of Interest Statement

The authors declare that the research was conducted in the absence of any commercial or financial relationships that could be construed as a potential conflict of interest.

